# Beyond grief: Quantifying bereavement needs of rural family caregivers

**DOI:** 10.1017/S1478951525100205

**Published:** 2025-09-05

**Authors:** Catherine Vanderboom, Diane Holland, Cory Ingram, Brystana G. Kaufman, Allison Gustavson, Jay Mandrekar, Ann Marie Dose, Ellen Wild, Carole Stiles, Joan M. Griffin

**Affiliations:** 1 Kern Center for the Science of Health Care Delivery Research, Mayo Clinic, Rochester, MN, USA; 2 Department of Community Internal Medicine Geriatrics, and Palliative Care, Mayo Clinic, Rochester, MN, USA; 3 Department of Population Health Sciences, Duke University School of Medicine, Durham, NC, USA; 4 Margolis Center for Health Policy, Duke University, Durham, NC, USA; 5 Durham Center for Innovation to Accelerate Discovery and Practice Transformation, Durham Veterans Affairs Health Care System, Durham, NC, USA; 6 Center for Care Delivery & Outcomes Research, Minneapolis Veterans Affairs Health Care System, Minneapolis, MN, USA; 7 Department of Medicine, University of Minnesota, Minneapolis, MN, USA; 8 Division of Biomedical Statistics and Informatics, Mayo Clinic, Rochester, MN, USA; 9 Division of Health Care Delivery Research, Mayo Clinic, Rochester, MN, USA

**Keywords:** Palliative care, bereavement support, family caregivers, rural, seriously illness, quality of life, social support, grief

## Abstract

**Objectives:**

Family caregivers (FCGs) may experience numerous psychosocial and practical challenges with interpersonal relationships, mental health, and finances both before and after their care recipient (CR) dies. The specific challenges affecting rural FCGs who often have limited access to palliative care services, transitional care, and other community resources are not well understood. The purpose of this paper is to quantify the challenges rural FCGs experienced immediately before the death of a CR and continuing into the bereavement period.

**Methods:**

A secondary analysis of data from a randomized controlled trial was conducted. The 8-week intervention included video visits between a palliative care research nurse and FCGs caring for someone with a life-limiting illness. Data were from structured interviews during nurse visits with FCGs in the intervention arm whose CR died during the intervention period.

**Results:**

Ninety (41.8%) of the 215 FCGs experienced the death of their CR. The majority of FCGs were female (58.9%), White (97.5%), spouses or partners (55.6%) and lived with the CR (66.7%). Most FCGs (84%) continued with intervention visits by the study nurse after the CR’s death. Visits resumed on average 7.2 days post-death. The majority of FCGs experienced challenges with grief/coping skills (56%) and interpersonal relationships/support systems (52%) both pre- and post-death of the CR. FCGs also experienced practical challenges with income/finance, housing, and communication with community resources both pre-and post-death.

**Significance of results:**

Bereavement support should extend beyond a focus on grief to include practical challenges experienced by FCGs. Because challenges experienced in the bereavement period often begin before a CR’s death, there is benefit in continuity of FCG support provided by a known clinician from pre- to post-death. When given an option, many rural FCGs are open to bereavement support as early as a week after the death of a CR.

## Background

Bereavement is the experience of losing a loved one and typically refers to the period immediately following death (Matthys et al. 2022). Bereavement can be associated with negative physical outcomes, such as worsening health, and changes in social and emotional health, including decreased satisfaction and well-being, loneliness, and social isolation (Shear et al. 2013).

Challenges experienced by family caregivers (FCGs) in the bereavement period often begin in the pre-death period (Diolaiuti 2021). FCGs face challenges of limited support, their own poor health, demanding family circumstances, and scarce personal time (Hudson et al. 2004). Caring for seriously ill individuals is often associated with increased caregiver burden and depression, decreased coping skills, and decreased quality of life (Haley et al. 2020; Le and Ibuka 2023). After a CR’s death, the day-to-day physical care demands subside but can be replaced with emotional, social, and practical challenges associated with transitioning away from caregiving.

Bereavement challenges are especially difficult for rural FCGs who often have limited access to palliative and transitional care services and other community resources (RAISE Family Caregiving Act 2022). Although community-based palliative care services are beginning to be available in rural areas, palliative care is still primarily confined to urban medical centers (Holland et al. 2020). Despite having more health problems, rural adults tend to use fewer health services because of limited availability of services and travel requirements (Agency for Healthcare Research and Quality 2021).

A variety of challenges are experienced by FCGs preceding and following the death of the CR. Grief is a commonly occurring emotional response before death (i.e., anticipatory grief) (Matthys et al. 2023; Skantharajah et al. 2022; Treml et al. 2021) and after death (Allen et al. 2021). In addition to grief, FCGs may have substantial mental health challenges, including difficulties with interpersonal relationships, mood changes, anxiety, and depression both pre- and post-death of a CR (Arias-Rojas et al. 2020). Practical challenges related to tangible assets, such as loss of income, a home, and financial security may complicate bereavement (Hospiscare n.d.).

Bereavement support commonly occurs after the death of a CR (Kirby et al. 2018) and often focuses primarily on grief. Effective bereavement support requires assessing and tailoring services to meet the needs of the bereaved (Hughes et al. 2019; Stroebe and Schut 2021). Providing support by interfacing with clinical social work staff during the immediate pre-death period can help the FCG’s adjustment by assisting them in preparing for emotional, social, and practical aspects of the CR’s death as well as the post-caregiving changes in their own life. Awareness of and support in preparing for the CR’s impending death is associated with improved FCG outcomes (Holtslander et al. 2017; Nielsen et al. 2016; Treml et al. 2021). In addition to grief and mental health challenges associated with loss, FCGs often face a broad range of practical decisions and tasks such as accessing funds, paying bills, or learning how to sell a house that are completed usually after the death of a loved one (Kirby et al. 2018). Kirby reported that most FCGs felt that standardized bereavement support did not include help with these types of challenges (Kirby et al. 2018).

The World Health Organization has long supported care for all bereaved caregivers (World Health Organization 2017). In the United States, Medicare requires hospices to provide bereavement services to family and friends for at least a year after the death of the patient (Center for Medicare and Medicaid Services Manual System 2011). Although Medicare has issued guidelines for providing bereavement services, there are no rules or regulations that specify which bereavement services should be provided, when, or by whom (Ghesquiere et al. 2019). For many hospices it is within the role of the clinical staff to provide pre-death support services, with designated bereavement staff providing support following death (National Archives and Records Administration 2023). FCGs consider bereavement team members’ collaboration with clinical team members pre-death to have a positive effect on FCG emotional well-being after the death of the CR (Matthys et al. 2023). Hospice interdisciplinary team meetings often provide communication between the clinical and bereavement staff.

The purpose of this secondary analysis is to quantify the bereavement-related challenges experienced by rural FCGs immediately pre- and post-death of a CR. Identifying FCG needs pre- and post-death is critical to designing, implementing, and scaling effective bereavement interventions that promote FCG well-being and inform best practices and guidelines for FCG support.

## Methods

Data are from a randomized controlled trial (RCT), the Technology-enhanced Transitional Palliative Care for Family Caregivers trial. This study evaluated the effect of a tailored video visit intervention designed to support FCGs who were caring for CRs eligible for palliative care during the transition from hospital to home. The study was designed to improve continuity of FCG care by providing teaching, guidance, and counseling (TGC) based on evidence-based transitional and palliative care principles that enhance FCGs’ caregiving knowledge and skills while also attending to FCGs’ own health and well-being. Among FCGs receiving the intervention were those whose CR died after hospital discharge during the 8-week intervention period.

The RCT details are described elsewhere (Holland et al. 2020). In summary, adult FCGs living in rural areas were recruited into the study during the hospitalization of adult patients with serious life limiting illnesses who were receiving palliative care in one of three states in the upper Midwest. FCGs were broadly defined as persons who self-identified as an unpaid caregiver for a palliative care patient. Once the patient was discharged from the hospital, a nurse initiated the intervention using video visits with the FCG. Study interventions were provided by experienced palliative care nurses who conducted interviews that included in-depth assessments utilizing a comprehensive list of potential problems. These problems were then addressed with TGC. TGC interventions were nursing activities that provided information about identified problems, encouraged actions and responsibility for self-care and coping, and assisted with decision making and problem solving with the FCG (Martin et al. 2011).

The protocol was designed for study visits to continue regardless of whether the CR was receiving hospice services. If a CR died, the study nurses would learn about the death by a call from the FCG, or if the FCG failed to connect for a study visit, the nurse would call the FCG. The study nurse offered to continue supporting the FCG after the death until the end of the 8-week intervention period, or earlier if the FCG chose to withdraw from the study. The FCGs were allowed to determine the timing of the post-CR death visits. FCGs’ and CRs’ sociodemographic data and basic health status were recorded at baseline.

As part of the intervention, challenges the FCGs experienced prior to and immediately following the death of the CR were documented after each visit by the study nurses in a separate, cloud-based electronic health record (EHR) created specifically for the FCG (Griffin et al. 2023). This EHR utilized the Omaha System, a research-based taxonomy that provides a standardized approach for assessment, documentation of interventions, and evaluation of health and social challenges (Nightingale Notes, Champ Software, Mankato, Minnesota, USA) (Holland et al. 2017; Martin et al. 2011). The Omaha System defines health and social challenges using a comprehensive list of 42 concepts labeled as problems. Each problem has a unique definition and set of signs and symptoms. Targets are provided that further define the specific focus within a problem. Specific problems and targets were identified by the study nurse assessments using all 42 Omaha System concepts, which were confirmed with the FCG during each study visit. The subsequent TGC interventions were based on the nurse’s assessment. There are detailed descriptions of interventions to address each problem/target dyad within the Omaha system (Martin et al. 2011).

For this analysis we included FCGs’ problems/targets as challenges that the study nurses intervened with by utilizing TGC during study visits. The study nurse may have provided guidance about dealing with the problem of “grief” by targeting “support systems” as one intervention. The guidance on developing support systems included ways to enhance interactions between family members and/or community resources. Teaching an FCG about the importance of rest and sleep (the target) while grieving (the problem) would have been another separate intervention. Counseling on income (the problem) may have included strategizing on how to pay bills, deal with insurance, and utilize family and community resources (the targets). Although labeling health and social challenges as problems may not be ideal, the ability to document needs-based interventions in a structured format allows researchers to compare interventions across predetermined categories (Griffin et al. 2023). By using a separate EHR, we were able to maintain a record for the FCGs that was independent of the CRs’ EHR. Study activities occurred between 2018 and 2022. The trial and study activities were registered at Clinicaltrials.gov (NCT03339271 Protocol version: 11) and were approved by the health care system’s Institutional Review Board (IRB# 17–005188).

## Results

Table 1 presents FCGs’ and CRs’ sociodemographic characteristics. The majority of FCGs were female (58.9%) and White (97.5%). Most were spouses/significant others (55.6%) or a child of the CR (33.3%) and lived with the CR (66.7%). Even though 92.4% of FCGs reported their health status as good or better prior to the intervention, at baseline 65% reported symptoms of depression (CESD score ≥ 10). Ninety of the 215 FCGs in the intervention group (41.8%) experienced the death of their CR after hospital discharge during the 8-week intervention period. Eighty-seven FCGs (97%) had at least one visit by the study nurse prior to the death of the CR. Seventy-six (84%) continued with visits after the death of their CR.

**Table 1.** FCG and CR sociodemographic data (*n* = 90)

FCG		*n* (%)
Gender	Male	37 (41.1)
	Female	53 (58.9)
Age, years	<45	16 (18.6)
	45−64.99	42 (48.8)
	65−69.99	15 (17.4)
	≥70	13 (15.1)
	Missing	4
Race	White	78 (97.5)
	Non-White	1 (1.3)
	>1 Race	1 (1.3)
	Missing	10
Self-reported health status	Fair	6 (7.6)
	Good	33 (41.8)
	Very good	31 (39.2)
	Excellent	9 (11.4)
	Missing	11
Caregiver CESD depression score on enrollment	<10	28 (35.0)
	≥10	52 (65.0)
	Missing	10
FCR relationship to CR	Spouse/Significant other	50 (55.6)
	Parent	30 (33.3)
	Child	3 (3.3)
	Other Relative	7 (7.8)
FCG lives with CR	No	30 (33.3)
	Yes	60 (66.7)
CR		(*N* = 90)
Gender	Male	39 (43.3)
	Female	51 (56.7)
Age	<45	9 (10.0)
	45−64.99	21 (23.3)
	65−69.99	9 (10.0)
	≥70	51 (56.7)
Race	White	82 (98.8)
	Non-White	1 (1.2)
	Missing	7
Hospital discharge disposition	Home	53 (58.9%)
	Facility	20 (22.2%)
	Other	17 (18.9%)
Time to death after hospital discharge	≤7 days	33 (36.7%)
	>7 days	57 (63.3%)

FCG = Family caregiver.

CR = Care recipient.

CESD = Center for Epidemiological Studies-Depression.

Most CRs were female (56.7%) and age 70 or greater (56.7%). The majority (58.9%) of CRs were discharged from the hospital to home in the community while less than a quarter (22.2%) were discharged to a care facility. Among the CRs, the majority (63.3%) lived for at least 7 days after hospital discharge.

Table 2 presents the number of study visits and TGC interventions. *Prior* to the death of their CR, FCGs received on average 4.9 visits (SD 3.2) with an overall average of 21.8 TGC interventions (SD 12.1) addressing identified challenges. *After* the death of their CR, each FCG received an average of 2.9 visits (SD 2.0) with an overall average of 11.0 TGC interventions (SD 6.2). Eighty-four percent of FCGs chose to resume visits on average 7.2 days (SD 5.8) after the death of their CR.

**Table 2.** Characteristics of intervention visits pre- and post-death

Number of visits prior to death of CR	N	87
	Mean (SD)	4.9 (3.2)
	Median	4.0
	Q1, Q3	2.0, 7.0
	Range	(1.0, 13.0)
Number of interventions prior to death of CR	N	87
	Mean (SD)	21.8 (12.1)
	Median	22.0
	Q1, Q3	11.0, 30.0
	Range	1−55
Number of visits after death of CR	N	76
	Mean (SD)	2.9 (2.0)
	Median	2.0
	Q1, Q3	1, 4
	Range	(1.0−8.0)
Date of 1st visit post-death	N	76
	Mean (SD)	7.2 (5.8)
	Median	8.0
	Q1, Q3	1.5, 12.0
	Range	(0.0−18.0)
Number of interventions post-death	N	76
	Mean (SD)	11.0 (6.2)
	Median	10.5
	Q1, Q3	6.5, 13.5
	Range	(1.0−25.0)

FCG = Family caregiver.

CR = Care recipient.

CESD = Center for Epidemiological Studies-Depression.

SD = standard deviation.

**Figure 1.**
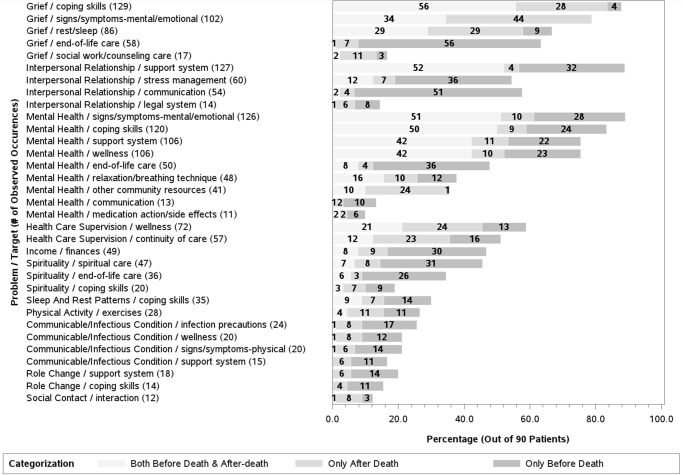
Frequency of family caregiver problems and targets during visits before and after care recipient death.

Figure 1 displays the types and number of TGC interventions that addressed FCG challenges (problems/targets). The study nurses intervened with the FCGs on 11 problems that included 33 unique problem/target challenges (as defined by the Omaha System) both pre- and post-CR death. Psychosocial challenges experienced by FCGs both pre-and post-CR death included grief, interpersonal relationships, and mental health. Interventions that addressed grief/coping skills occurred 129 times during the study and transpired in both pre- and post-CR death for 56% of the FCGs. Interventions that addressed interpersonal relationship/support systems occurred 127 times during the study. Approximately half of the FCGs (52%) received interventions to address interpersonal relationship/support systems challenges both pre- and post-CR death. Interventions that addressed mental health signs and symptoms occurred 126 times during the study and in both pre-and post-CR death for 51% of the FCGs.

Practical challenges were also addressed pre- and post-CR death and included income, sleep and rest patterns, and physical activity. Interventions that addressed income occurred 49 times during the study and occurred in both pre- and post-CR death for 8% of the FCGs. Interventions that addressed sleep and rest patterns occurred 35 times during the study and were reported in both pre- and post-CR death for 9% of the FCGs. Interventions that addressed physical activity occurred 28 times in both pre- and post-CR death for 4% of the FCGs.

Challenges related to following protocols for communicable and infectious conditions associated with the COVID-19 pandemic were also documented (Holland et al. 2021). Caregiving challenges with associated target areas occurred frequently for FCGs prior to CR death. Not surprisingly, after the death of the CR, FCGs did not report any ongoing challenges associated with caregiving (data not shown).

## Discussion

This study extends the existing literature by quantifying the challenges experienced by rural FCGs and addressed by TGC interventions prior to and immediately following the death of a CR. Using quantitative data, our study found specific problems experienced by rural FCGs that have not previously been identified. FCGs experienced a range of emotional, social, and practical challenges prior to the death of their CR, and many of the challenges continued into the bereavement period. These findings align with Harrop et al. (2021) who, while not distinguishing between rural and urban FCGs, espoused that the main objectives of bereavement services were to support the FCG’s experience of grief by addressing their quality of life, mental wellbeing, and ability to cope. Stroebe and Schut (2021), who also did not distinguish between rural and urban participants, supported the idea that beyond dealing with the stressful, emotional aspects of the loss itself, other FCG challenges were associated with transitioning away from caregiving and must be dealt with because of the life changes that occur after caregiving.

In a meta-summary of qualitative studies, Holtslander et al. (2017) reinforced the need for various kinds of support during bereavement, including support for practical life challenges. In our study, we quantified the specific kinds of pre- and post-death practical support needed by our sample of FCGs, including financial and self-care issues. Helpful practical assistance included tailored interventions that were focused on mutually identified problems, such as the FCG’s physical activity, sleep, and rest. Assistance with practical challenges, such as with income and other financial issues that complicate the bereavement process, are also warranted and should be assessed during the pre-death bereavement period and addressed by palliative care teams, including clinical social work staff when possible (Ham et al. 2022).

The majority of FCGs in this study requested to continue study visits as early as 1 week after the death of the CR, indicating the perceived value of continuing their relationship with the intervention nurse in addition to other post-death interactions with family and friends. This was likely because they already had an established relationship with the intervention nurse prior to the CR death. McGinley and Waldrop (2020) and Sawyer et al. (2021) both affirm that continuity of care is key for effective communication in bereavement and allows for sustained support.

Several qualitative studies have also found that a continuous relationship between the FCG and member(s) of the care team pre- and post-death is less stressful for FCGs and provides for timely and consistent bereavement care (Aoun et al. 2017; Hughes et al. 2019; Tabler et al. 2015). Similar to the visits received by FCG in our study, Tabler reported that FCGs preferred 3 to 5 bereavement visits with known staff after the CR’s death to discuss issues applicable to them (Tabler et al. 2015).

## Limitations

There are several limitations to this study which may influence the interpretation of the results. First, this was a secondary analysis of existing data. Second, all participants resided in resource-constrained rural settings in the upper Midwest of the United States, and the results may not be applicable to individuals living in other locales where resources are more readily available. Third, the majority of the FCGs were White and college educated, which may limit the generalizability of the findings to more diverse populations. Lastly, the study protocol and subsequent analysis did not differentiate between individuals receiving or not receiving hospice services, although the study intervention did not vary based on whether hospice was involved. Nevertheless, this study contributes quantitative data to the body of knowledge regarding appropriate services for rural FCGs before and after the death of a CR.

## Clinical implications

While there is a growing body of literature describing the transitional care needs of FCGs during pre- and post-death of the CR (Coleman 2016; Leighton et al. 2020; Levoy et al. 2022), the purpose of this analysis was to provide quantitative data about challenges experienced by rural FCGs. We found the majority of FCGs welcomed continued care from the interventionists following the death of their CR. Palliative care nurse interventionists provided TGC interventions related to a variety of psychosocial issues and practical challenges of concern to FCGs and had the benefit of consulting with other team members, including social workers. Our results indicate the importance of bereavement support provided by individuals who are experts at connecting with community services to address practical needs, such as finances, selling a home, and mobilizing social support. These experts can help reinforce the idea that community-based bereavement services must encompass services beyond grief support. Our results also suggest that palliative care social workers working in tandem with grief support team members during the bereavement period may provide an effective solution for addressing rural FCG needs.

## Implications for future research

Our methodology and documentation system allowed for data collection at the time of the nurse visits rather than retrospectively. This approach to data collection is quite different than several studies in which data were collected months after the death of the CR (Aoun et al. 2018, 2017; Ham et al. 2022; Matthys et al. 2023). The FCG’s data were maintained in a EHR separate from the CRs’ EHR, which facilitated data collection.

A gap continues in the knowledge base that quantifies rural FCG pre- and post-death challenges. Data in this study from rural FCGs allows future studies to distinguish differences in bereavement needs experienced by FCGs living in rural versus urban areas. These findings also indicate the value of broad-based bereavement support by a known and engaged care provider before the death of the CR and during the immediate bereavement period. Future research is warranted to develop and test approaches to care that provide actionable plans for assessing and providing a broader array of bereavement support services that recognize the diverse challenges faced by rural FCGs and the value of continuity of care.
